# A New Medical Journal

**Published:** 1853-10

**Authors:** 


A NEW MEDICAL JOURNAL.

"The cry is still, they come!" We have received the following prospectus of a new medical journal, to be issued under the auspices of the Medical Society of Virginia:

"The following scheme is proposed, but will, of course, be subject to modification by the Society:

"The *Journal* shall be edited by a corps of seven competent individuals, to be chosen annually by the State Medical Society. It shall appear Quarterly—in January, April, July, and October — each number containing one hundred and ninety-two pages, making, at the end of the year, a volume of seven hundred and sixty-eight pages.

"To each of the editors a particular department of medicine shall be assigned, and no article shall appear in its columns which is not approved by all.

"By this arrangement the subscribers will have a satisfactory guarantee that no inferior matter will ever disgrace the work, but that they may look to it with confidence for information on which they may rely.

"The price of subscription will be *contingent on the number of subscribers,* but will in no case exceed *four dollars per annum, in advance.*"

Two or three remarks are suggested by this scheme. In the first place, we cannot see, at this distance, why the Medical Society of Virginia should have passed by both the medical journals now in progress in Richmond. It occurs to us that one or the other of these excellent periodicals might have been made the vehicle for communicating their transactions; but there are doubtless reasons influencing the Society of which we are not apprised. We cannot, however, withhold the opinion that there is some danger of the business of medical journalism being overdone in the Old Dominion. But if the new journal must be added to the long list of our serials, we suggest to the Society that the number of editors proposed is too large. They will never be able to co-operate. Seven doctors to meet regularly and consult about the details of a medical quarterly! The thing seems to us wholly impracticable. "The half is better than the whole," is a maxim of one of the ancients, which will certainly be found to be true in this instance if we are not greatly mistaken. There can be no doubt if the scheme is carried into execution, that the journal will be an able one. We only question the policy of dividing the professional mind of the State, instead of concentrating its force upon the journals already in existence.

